# From Genes to Clinical Practice: Exploring the Genomic Underpinnings of Endometrial Cancer

**DOI:** 10.3390/cancers17020320

**Published:** 2025-01-20

**Authors:** Thulo Molefi, Lloyd Mabonga, Rodney Hull, Motshedisi Sebitloane, Zodwa Dlamini

**Affiliations:** 1 Discipline of Obstetrics and Gynaecology, School of Clinical Medicine, University of KwaZulu-Natal, Durban 4002, South Africa; thulo.molefi@up.ac.za; 2 SAMRC Precision Oncology Research Unit (PORU), DSI/NRF SARChI Chair in Precision Oncology and Cancer Prevention (POCP), Pan African Research Institute (PACRI), University of Pretoria, Hartfield, Pretoria 0028, South Africa; lloyd.mabonga@up.ac.za (L.M.); rodney.hull@up.ac.za (R.H.); 3 Department of Medical Oncology, University of Pretoria, Hatfield, Pretoria 0028, South Africa

**Keywords:** endometrial cancer, genetic alterations, molecular subtypes, CRISPR, multi-omics, personalized medicine, precision oncology, targeted therapies

## Abstract

Endometrial cancer (EC), a prevalent gynecological malignancy, presents significant challenges due to its genetic complexity and heterogeneity. The genomic landscape of EC is underpinned by genetic alterations, such as mutations in PTEN, PIK3CA, and ARID1A, and chromosomal abnormalities. The identification of molecular subtypes—POLE ultramutated, microsatellite instability (MSI), copy number low, and copy number high—illustrates the diverse genetic profiles within EC and underscores the need for subtype-specific therapeutic strategies. The integration of multi-omics technologies such as single-cell genomics and spatial transcriptomics has revolutionized our understanding and approach to studying EC and offers a holistic perspective that enhances the ability to identify novel biomarkers and therapeutic targets. The translation of these multi-omics findings into personalized medicine and precision oncology is increasingly feasible in clinical practice. Targeted therapies such as PI3K/AKT/mTOR inhibitors have demonstrated the potential for improved treatment efficacy tailored to specific genetic alterations. Despite these advancements, challenges persist in terms of variability in patient responses, the integration of genomic data into clinical workflows, and ethical considerations. This review explores the genomic underpinnings of EC, from genes to clinical practice. It highlights the ongoing need for multidisciplinary research and collaboration to address the complexities of EC and improve diagnosis, treatment, and patient outcomes.

## Simple Summary

Endometrial cancer is becoming more common, and current treatments do not work well for everyone. The study aims to understand how genetic changes drive this type of cancer and how these insights can improve treatment. It explores key genetic mutations and how they influence the development of cancer, with the goal of helping to classify the disease more precisely and design targeted therapies that are tailored to individual patients. By connecting genetics to clinical care, this research could lead to earlier diagnoses, better treatment options, and improved survival rates. It also sets the stage for future studies, giving the scientific community a clearer roadmap to enhance cancer care.

## 1. Introduction

Endometrial cancer (EC), a malignancy originating from the lining of the uterus, represents a significant public health concern due to its prevalence and impact on women’s health worldwide [1]. It is the most common gynecologic cancer in developed countries and ranks second after cervical cancer in developing regions [2]. The increasing incidence of EC, particularly in high-income countries, underscores the urgent need for effective prevention, early detection, and treatment strategies [3]. EC is characterized by its high incidence and significant mortality, especially among postmenopausal women. According to the World Health Organization (WHO), the incidence of EC varies globally, with higher rates observed in North America, Europe, and Oceania, and lower rates in Asia and Africa [4]. In the United States, EC is the fourth most common cancer in women, with an estimated 65,620 new cases and 12,590 deaths projected in 2020 [5]. In 2024, the estimated number of deaths due to EC in the United States is estimated to be 13,250. This represents a significant proportion of cancer-related mortalities among women and underscores the disease’s impact as a leading cause of cancer death in females. EC accounts for 3.4% of all new cancer cases, and it is the thirteenth leading cause of cancer-related deaths in the country [6]. In Europe, the incidence of EC is notably high, particularly in countries with aging populations and elevated obesity rates [1].

**Figure 1:**
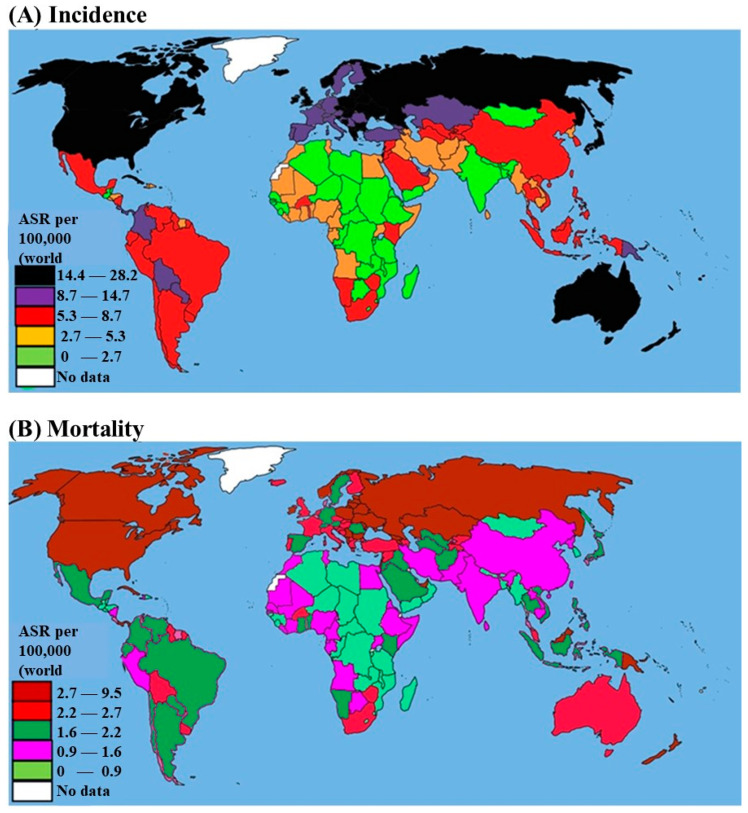
Worldwide incidence and mortality rates for endometrial cancer: (**A**) Europe, North America, and Oceania have the highest incidence rates, while Southeast and Central Asia, Latin America, and Africa have the lowest rates. (**B**) However, the mortality rate is disproportionality higher in these lower-income regions, due to limited access to early diagnostic services and effective treatment options [4].

Conversely, in low-income countries, while the incidence is lower, mortality rates are higher due to limited access to early diagnostic services and effective treatment options [7]. Historically, the incidence rates in African countries have been lower than in Western nations. However, recent trends indicate a rise in cases attributed to factors such as urbanization, lifestyle changes, and increasing obesity rates [8]. According to Bray and co-workers, the age-standardized incidence rate (ASIR) of EC in Africa is approximately 4.3 per 100,000 women, which is lower than the global average of 8.4 per 100,000 women [9]. Despite these lower incidence rates, significant variation exists across the continent, with higher rates reported in North Africa compared to in Sub-Saharan Africa **(**Figure 1) [4]. Several factors contribute to the rising incidence of EC. These include increasing life expectancy, the prevalence of obesity, and the widespread use of unopposed estrogen therapy [1]. Obesity is a particularly significant risk factor, as it is associated with increased estrogen levels due to the aromatization of androgens in adipose tissue, leading to endometrial hyperplasia and, subsequently, cancer [10]. Other risk factors include early menarche, late menopause, nulliparity, and certain genetic predispositions such as Lynch syndrome [3].

The impact of EC on women’s health extends beyond mortality and morbidity, affecting quality of life, reproductive health, and socioeconomic status [1]. The disease often presents with symptoms such as abnormal uterine bleeding, pelvic pain, and weight loss, which can lead to significant psychological distress and physical discomfort [11]. EC and its treatments, including surgery, radiation, and chemotherapy, can lead to long-term side effects such as sexual dysfunction, lymphedema, and fatigue. These complications adversely affect the quality of life of survivors, necessitating comprehensive survivorship care plans that address physical, emotional, and social needs [12]. For premenopausal women, EC poses a substantial threat to fertility. Standard treatment often involves hysterectomy, which precludes the possibility of future pregnancies. Fertility-sparing treatments, such as progestin therapy, are available but are appropriate only for select patients with early-stage, low-grade tumors [13]. This highlights the need for early diagnosis and the development of less invasive treatment options. The socioeconomic impact of EC is profound, particularly in low- and middle-income countries (LMICs) where healthcare resources are limited. Women in these regions often present with advanced disease due to lack of access to screening and diagnostic services, resulting in higher treatment costs and poorer outcomes [7]. Moreover, the financial burden of cancer treatment can lead to catastrophic health expenditures, further exacerbating poverty and inequality.

Despite advancements in diagnosis and treatment, significant disparities exist in the burden of EC globally. Women in high-income countries benefit from better healthcare infrastructure, access to advanced diagnostic tools, and effective treatments, resulting in higher survival rates. In contrast, women in LMICs face numerous barriers, including limited awareness, inadequate healthcare facilities, and financial constraints, leading to delayed diagnosis and suboptimal treatment outcomes [8]. Efforts to reduce the global burden of EC must focus on addressing these disparities through international collaboration, capacity building, and the implementation of cost-effective screening and treatment programs. Public health initiatives aimed at reducing obesity, promoting healthy lifestyles, and increasing awareness about the symptoms and risk factors of EC are crucial in mitigating its impact [3].

Thus, EC remains a significant public health challenge with considerable implications for women’s health globally. Its rising incidence, particularly in high-income countries, coupled with its profound impact on quality of life, reproductive health, and socioeconomic status, underscores the need for comprehensive strategies to improve prevention, early detection, and treatment [1]. Addressing global disparities through targeted interventions and international cooperation is essential in reducing the burden of this disease and enhancing outcomes for women worldwide [3].

## 2. Importance of Genomic Studies in Cancer

Genomic studies have profoundly transformed the landscape of cancer research and treatment, ushering in an era of precision medicine where therapies are tailored to the genetic profiles of individual tumors [14]. This approach has been particularly impactful in understanding and treating EC, which has diverse molecular subtypes with distinct prognoses and therapeutic responses [15]. Genomic studies have revealed that cancers are not homogeneous but consist of multiple subtypes with unique genetic and molecular characteristics. Identifying specific genetic mutations and pathways involved in cancer has led to the development of targeted therapies [14]. For instance, the discovery of mutations in the PI3K/AKT/mTOR pathway in EC has spurred the development of targeted inhibitors, improving treatment outcomes for patients with these genetic alterations [2]. Several FDA-approved targeted inhibitors have been integrated into the treatment landscape for EC, leveraging the molecular characteristics of tumors to improve outcomes. Pembrolizumab (Keytruda) is an immune checkpoint inhibitor targeting PD-1, while Lenvatinib (Lenvima) is a multi-tyrosine kinase inhibitor affecting angiogenesis and tumor growth [16]. The combination is approved for advanced or metastatic endometrial carcinoma that is not MMR-deficient or MSI-high, after progression following systemic therapy. The KEYNOTE-775/Study 309 trial demonstrated a significant improvement in progression-free survival (7.2 vs. 3.8 months) and overall survival (18.3 vs. 11.4 months) among patients with advanced EC compared to chemotherapy [16].

Dostarlimab is an anti-PD-1 antibody used for tumors with dMMR or MSI-high profiles. It is approved for dMMR recurrent or advanced endometrial cancer in patients progressing after platinum-based chemotherapy. Based on the GARNET trial, which showed a 43.5% objective response rate (ORR) for dMMR patients, including durable responses lasting beyond six months in many cases [17]. Trastuzumab targets HER2 overexpression, a feature present in a subset of endometrial cancers, particularly serous carcinomas. While not being specifically FDA-approved for endometrial cancer, it has shown efficacy in HER2-positive endometrial cancer when combined with chemotherapy [18]. The GOG 86P trial demonstrated improved outcomes in HER2-positive tumors when trastuzumab was added to chemotherapy [19]. Everolimus is an mTOR inhibitor that disrupts cell proliferation and survival pathways. While not exclusively approved for endometrial cancer, it has shown efficacy in combination with letrozole in hormone receptor-positive cases. Studies indicate improved disease control in recurrent or metastatic endometrial carcinoma with this combination [19]. Future approvals underscore the shift toward precision medicine, driven by molecular profiling and biomarker identification. Emerging therapies, including antibody–drug conjugates and novel inhibitors, are poised to expand the therapeutic armamentarium.

Genomic studies have facilitated the use of immunotherapy in cancer treatment for tumors with high mutational burden, such as the MSI-H subtype of EC [16,18]. Recent FDA approvals of immunotherapy for EC were based on pivotal clinical trials demonstrating the efficacy of combination therapies involving immune checkpoint inhibitors. The KEYNOTE-868/NRG-GY018 trial assessed pembrolizumab, an immune checkpoint inhibitor, combined with chemotherapy (carboplatin and paclitaxel) in patients with advanced or recurrent EC [20]. Results showed a significant improvement in progression-free survival, particularly in patients with mismatch repair-deficient (dMMR) tumors. In this group, the median progression-free survival was not reached for the pembrolizumab arm compared to 6.5 months for the placebo arm, highlighting the potential of this therapy to delay cancer progression. Similar benefits were observed in mismatch repair-proficient (pMMR) patients, although to a lesser degree [20]. Durvalumab, another checkpoint inhibitor, was evaluated in combination with chemotherapy in advanced or recurrent endometrial cancer in the DUO-E trial [21]. While the trial revealed significant progression-free survival benefits across all patients, the strongest effects were noted in dMMR patients. Median progression-free survival in this subgroup was not reached, compared to 7 months for the placebo arm [21].

Dostarlimab, used in combination with chemotherapy, demonstrated improved outcomes in dMMR and certain subsets of pMMR patients in the RUBY trial [22]. This trial further established the role of immunotherapy in tailoring treatments based on genetic markers. These approvals signify a shift towards personalized medicine in endometrial cancer, leveraging immune-based therapies for enhanced outcomes, particularly in genetically distinct subgroups [22]. The PHAEDRA clinical trial (phase 2, ANZGOG1601) also evaluated the efficacy and safety of durvalumab, an immune checkpoint inhibitor, in patients with advanced EC [23]. The study categorized participants based on mismatch repair (MMR) status: MMR-deficient (dMMR) or MMR-proficient (pMMR). Patients received durvalumab as monotherapy after 0–3 prior lines of chemotherapy [23]. Among dMMR patients, the ORR was 47%, with complete or partial responses observed. In contrast, pMMR patients showed a significantly lower ORR of 3%. Median progression-free survival (PFS) was 5.5 months for dMMR and 1.8 months for pMMR patients. The median overall survival (OS) was not reached for dMMR patients, whereas pMMR patients had a median OS of 11.5 months [23]. Durvalumab demonstrated significant efficacy in dMMR EC, making it a promising treatment for this subset, but its impact on pMMR tumors was limited. The results highlight the importance of molecular profiling in treatment selection for EC [23].

Genomic studies have identified biomarkers that can predict disease prognosis and treatment response. For EC, biomarkers such as mutations in PTEN, PIK3CA, and ARID1A are associated with disease progression and survival outcomes [24]. Advances in genomic technologies have enabled the development of liquid biopsies, which detect cancer-related genetic mutations and circulating tumor DNA (ctDNA) in blood samples. This non-invasive method holds promise for early cancer detection, monitoring disease progression, and assessing treatment response [25]. Genomic studies have elucidated mechanisms underlying resistance to conventional therapies. For example, alterations in the FGFR2 gene have been implicated in resistance to hormonal therapies in EC, leading to the exploration of FGFR inhibitors as a potential therapeutic option [26]. The advent of NGS has revolutionized genomic studies, allowing comprehensive profiling of tumor genomes. This technology has enabled the identification of novel genetic alterations and the development of multigene panels for cancer diagnosis and treatment selection [27].

EC is a prime example of how genomic studies have advanced our understanding and treatment of cancer. The classification of EC into molecular subtypes has provided insights into tumor behavior and guided treatment decisions [28]. For instance, patients with POLE-ultramutated tumors have an excellent prognosis and may not require aggressive treatment, whereas those with copy-number-high tumors often benefit from more intensive therapy [18,29]. Genomic studies have identified actionable mutations in EC, leading to the development of targeted therapies. The identification of mutations in the PI3K/AKT/mTOR pathway has resulted in clinical trials testing inhibitors of this pathway, offering new hope for patients with advanced disease [2]. Genomic profiling of endometrial tumors enables personalized treatment approaches. For example, patients with MSI-H tumors are now being treated with immune checkpoint inhibitors, demonstrating the impact of genomics on improving patient outcomes [18]. The integration of genomic data into clinical practice is exemplified by the use of molecular markers for risk stratification and treatment planning in EC. This approach ensures that patients receive the most appropriate and effective therapies based on their tumor’s genetic profile [24].

Genomic studies have revolutionized cancer research and treatment, providing unprecedented insights into the molecular underpinnings of the disease. In EC, genomic advancements have led to improved classification, targeted therapies, and personalized medicine approaches, ultimately enhancing patient care and outcomes [15]. Continued investment in genomic research is essential to further unravel the complexities of cancer and develop innovative strategies to combat this devastating disease. A comprehensive understanding of the genomic landscape of EC is indispensable in bridging the gap between genetic findings and clinical applications.

## 3. Genomic Landscape of Endometrial Cancer

**Figure 2:**
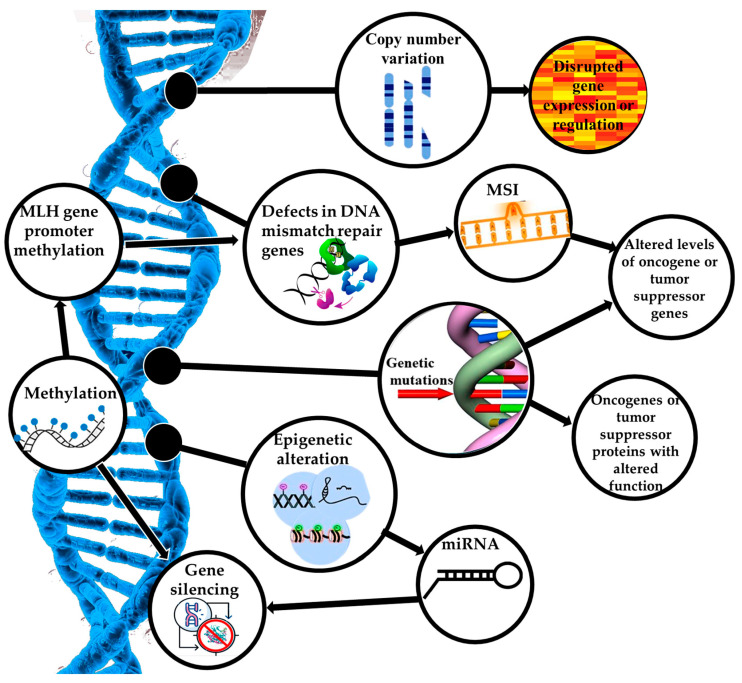
The genomic underpinnings of endometrial cancer. The pathogenesis of EC is a complex process of molecular mechanisms (large circles) involved in the manifestation of genetic mutations, activation of oncogenes, and inactivation of tumor suppressor genes (small circles). Defects in the DNA MMR pathway can lead to MSI and result in changes in the expression of various genes; this can result in imbalances in hormonal signaling pathways and changes in processes such as angiogenesis. Changes in gene expression and activity can also result from mutations and copy number variation. Epigenetic changes can result in gene silencing as a result of miRNA activity and methylation. MicroRNAs (miRNAs) play significant roles in the post-transcriptional regulation of gene expression and their dysregulation has been linked to various aspects of EC progression. Methylation of the promoters of genes such as *MLH* can affect the DNA MMR pathway.

EC is a heterogeneous disease characterized by a variety of genetic and epigenetic alterations. It is a prevalent gynecological malignancy with significant genetic heterogeneity. As shown in Figure 2, the pathogenesis of EC is a complex process of molecular mechanisms involved in the manifestation of genetic mutations, the activation of oncogenes and inactivation of tumor suppressor genes, defects in the DNA MMR pathway, an imbalance in hormonal signaling pathways, epigenetic changes, disorders in angiogenesis, and others. Understanding the genomic landscape of EC is crucial for improving diagnosis, treatment, and prognosis [30].

**Figure 3:**
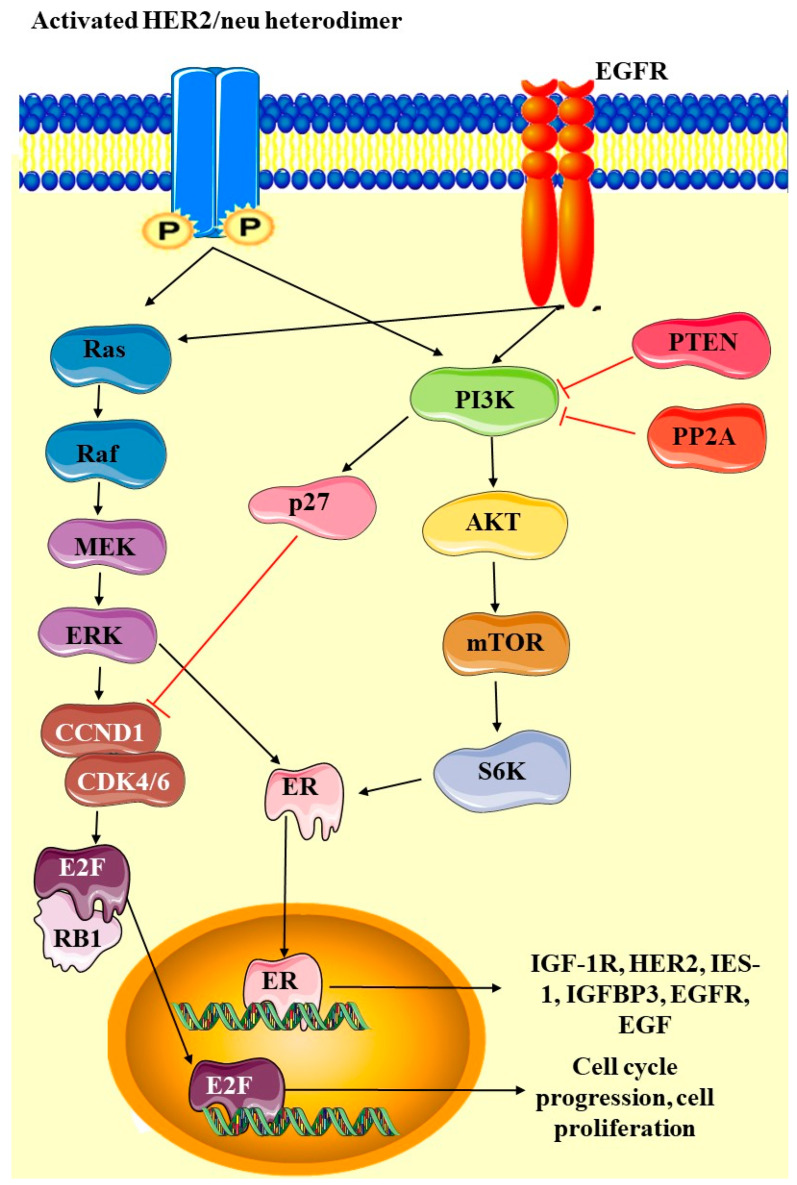
The PI3K-AKT-mTORc1 pathway is extensively involved in endometrial serous carcinoma carcinogenesis. The activation of the pathway begins with the stimulation of receptor tyrosine kinases (e.g., HER2 or IGF-1R) by their respective ligands, leading to phosphorylation of PI3K. Activated PI3K catalyzes the conversion of PIP2 to PIP3, initiating downstream signaling. Aberrations in this pathway, including mutations in PTEN, PIK3CA, and PIK3R1, or the activation of upstream receptor tyrosine kinases drives carcinogenesis by promoting uncontrolled cellular proliferation, survival, and growth. PTEN loss results in sustained PIP3 accumulation, persistent AKT activation, and hyperactivation of the pathway. AKT activation phosphorylates and inactivates pro-apoptotic proteins like BAD, leading to reduced apoptosis. It also enhances Cyclin D1 activity while inhibiting cell cycle inhibitors such as p21 and p27. AKT activates the mechanistic target of rapamycin complex 1 (mTORC1), a master regulator of protein synthesis and cellular metabolism. Through S6K and 4E-BP1, mTORC1 promotes ribosomal biogenesis and cap-dependent translation, driving tumor cell growth. Negative feedback via S6K phosphorylation of IRS-1 dampens upstream signaling, but this regulatory mechanism is often bypassed in cancer due to mutations. Abbreviations: HER, human epidermal growth factor receptor; EGFR, epidermal growth factor receptor; PI3K, phosphatidylinositol3 kinase; PTEN, phosphatase and tensin homolog; PP2A, protein phosphatase 2, regulatory subunit A, alpha; AKT, v-akt murine thymoma viral oncogene homolog 1; mTORc, mammalian target of rapamycin complex.

Among the various genetic alterations observed in EC, mutations in the PTEN (phosphatase and tensin homolog) gene are particularly noteworthy due to their high frequency and critical role in tumorigenesis [28,31]. Mutations in the *PTEN* gene are among the most common genetic alterations in EC, occurring in approximately 40–80% of cases, particularly in the endometrioid subtype which is more common and has a better prognosis compared to that of the non-endometrioid types [30,31]. It is a tumor suppressor gene located on chromosome 10q23, and it encodes a phosphatase involved in regulating the PI3K/AKT signaling pathway. *PTEN* mutations lead to loss of function and the activation of the PI3K/AKT pathway, thereby promoting and contributing to uncontrolled cell proliferation and survival, which are hallmarks of cancer (shown in Figure 3). These mutations can include point mutations, insertions, deletions, and frameshift mutations, leading to a loss of PTEN protein function [28,31].

The PTEN protein acts as a phosphatase, dephosphorylating PIP3 (phosphatidylinositol-3,4,5-triphosphate) to PIP2 (phosphatidylinositol-4,5-bisphosphate), thereby antagonizing the PI3K/AKT signaling pathway [31,32,33]. This pathway is crucial for cell survival, growth, and proliferation. Loss of PTEN function results in unchecked activation of AKT, promoting cellular processes that lead to tumorigenesis, such as increased cell proliferation, survival, and migration [31,33]. The presence of *PTEN* mutations in EC has significant clinical implications. Studies have shown that PTEN loss is associated with early-stage disease and favorable prognosis, particularly in endometrioid endometrial carcinoma (EEC) [34]. However, the prognostic significance of *PTEN* mutations can vary depending on the context of other genetic alterations and tumor subtypes. Additionally, PTEN status can influence therapeutic responses. For instance, tumors with PTEN mutations may be more sensitive to inhibitors targeting the PI3K/AKT/mTOR pathway, offering potential for targeted therapy in these patients. This has prompted ongoing research into PI3K pathway inhibitors and their efficacy in PTEN-mutant ECs [31,33,34]. PTEN mutations often result in increased activation of the PI3K/AKT pathway due to loss of negative regulatory control. This hyperactivation suggests that tumors with PTEN mutations may be particularly sensitive to therapies targeting this pathway [35]. Preclinical and clinical studies have provided evidence supporting this hypothesis. Studies using PTEN-deficient cancer cell lines and mouse models have shown increased sensitivity to PI3K inhibitors, such as buparlisib (BKM120) and alpelisib (BYL719) [36]. In PTEN-deficient EC cell lines, PI3K inhibitors significantly reduced cell viability and tumor growth, suggesting pathway dependence for survival and proliferation [37]. PTEN loss leads to compensatory signaling pathways, such as enhanced MAPK activity. Combining PI3K inhibitors with MEK inhibitors in PTEN-deficient models demonstrated synergistic effects, reducing cell viability more effectively than monotherapy [38].

The ALPINE phase II trial evaluated alpelisib, a PI3K-alpha-specific inhibitor, in solid tumors harboring PIK3CA mutations. While not exclusive to PTEN mutations, PTEN-deficient tumors showed notable responses, highlighting their dependence on PI3K signaling. In ECs specifically, alpelisib demonstrated efficacy in reducing tumor burden in patients with coexisting PTEN and PIK3CA alterations [39]. In the GARNET trial, PTEN-deficient endometrial cancers showed improved responses when combining immune checkpoint blockade (dostarlimab) with PI3K pathway inhibitors, further suggesting that targeting PI3K compensates for the heightened oncogenic signaling due to PTEN loss [17]. In the MATCH trial, PTEN-mutant tumors treated with PI3K inhibitors showed partial responses or disease stabilization, especially in endometrial and breast cancers. These results confirmed the viability of pathway-specific targeting based on molecular profiling [40]. However promising, the responses to PI3K inhibitors in PTEN-deficient tumors are heterogeneous. Compensatory pathways, such as upregulated mTOR signaling, necessitate combination approaches [35]. Additionally, toxicity from PI3K inhibitors, particularly hyperglycemia and rash, remains a barrier to broad application. Thus, PTEN mutations increase sensitivity to PI3K inhibitors, as demonstrated in preclinical and clinical studies. However, therapeutic success may require addressing compensatory mechanisms and optimizing combination regimens [17].

Recent research has focused on understanding the broader implications of *PTEN* mutations in the molecular landscape of EC [33]. For example, next-generation sequencing and comprehensive genomic profiling have revealed that *PTEN* mutations often coexist with other genetic alterations, such as mutations in *PIK3CA*, *ARID1A*, and *KRAS*, suggesting a complex interplay of genetic events driving endometrial carcinogenesis. Moreover, studies have explored the role of *PTEN* mutations in the context of immune response and tumor microenvironment [28]. Emerging evidence suggests that PTEN loss can affect immune cell infiltration and the expression of immune checkpoint molecules, thereby influencing the tumor’s response to immunotherapy [33]. Therefore, *PTEN* mutations play a crucial role in the pathogenesis of EC, particularly in the endometrioid subtype. Their high prevalence and significant impact on tumor biology underscore the importance of PTEN as both a biomarker and a potential therapeutic target [31]. Ongoing research continues to unravel the complexities of PTEN-related signaling pathways and their interactions with other molecular events, paving the way for more effective and personalized treatment strategies in EC [15].

**Figure 4:**
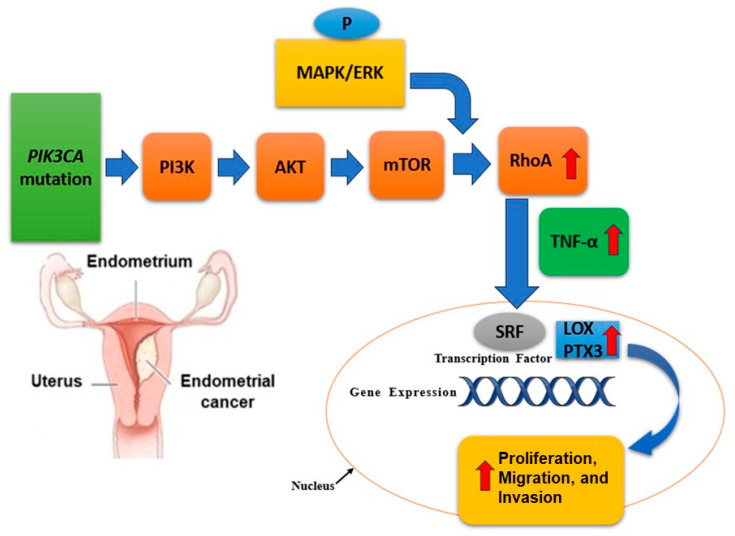
PIK3CA mutations in endometrial cancer. The presence of the PIK3CA mutation stimulates PI3K, phosphorylating PIP2 to PIP3 and activating mTOR and AKT. RhoA activity, which controls the serum response elements (SRFs) with the aid of TNF-α, is impacted by both mTOR and MAPK/ERK. Cell proliferation, migration, and invasion are caused by the transcription factor SRF’s downstream effects on LOX and PTX3.

Mutations in the *PIK3CA* gene, which encodes the p110α catalytic subunit of phosphatidylinositol-3-kinase (PI3K), are particularly significant and are present in about 40-50% of EC cases, with a higher frequency in endometrioid subtypes compared to in non-endometrioid subtypes [18]. These mutations play a crucial role in the activation of the PI3K/AKT/mTOR signaling pathway, contributing to tumorigenesis and progression in EC [32]. These mutations often occur in the helical (exon 9) and kinase (exon 20) domains of the gene, leading to gain-of-function alterations that enhance PI3K activity [18,29]. The PI3K/AKT signaling pathway is a critical regulator of cell growth, survival, and metabolism. As shown in Figure 4, *PIK3CA* mutations result in constitutive activation of this pathway, leading to increased cell proliferation, survival, and angiogenesis, which are hallmarks of cancer [41]. This hyperactivation occurs through the phosphorylation and activation of AKT, which then promotes the downstream signaling events crucial for tumor development and progression [14]. The presence of *PIK3CA* mutations in EC has several clinical implications. Some studies suggest that *PIK3CA* mutations are associated with a better prognosis in endometrioid endometrial carcinoma, though the results are not always consistent across different cohorts [32]. *PIK3CA* mutations have been targeted with specific inhibitors of the PI3K/AKT/mTOR pathway. For example, drugs like alpelisib (PIQRAY) have shown efficacy in PIK3CA-mutant cancers, including EC, making these mutations a potential biomarker for targeted therapy [42].

There is ongoing research into combining PI3K inhibitors with other treatments such as immune checkpoint inhibitors, given the role of the PI3K pathway activation in modulating the tumor immune microenvironment [43]. Recent advances in genomic profiling have enhanced our understanding of *PIK3CA* mutations in EC. Comprehensive genomic analyses, such as those conducted by The Cancer Genome Atlas (TCGA), have provided detailed insights into the co-occurrence of *PIK3CA* mutations with other genetic alterations, such as PTEN loss and ARID1A mutations, which collectively influence the disease phenotype and therapeutic response [18,29]. Moreover, emerging research is focusing on the interaction between *PIK3CA* mutations and other molecular pathways, such as the MAPK pathway, to identify novel combinatorial treatment strategies [29]. For instance, preclinical studies have demonstrated synergistic effects of PI3K and MEK inhibitors in *PIK3CA*-mutant EC models, highlighting potential new avenues for treatment [18]. Thus, *PIK3CA* mutations are a significant driver of EC, particularly in the endometrioid subtype. These mutations lead to the activation of the PI3K/AKT signaling pathway, promoting tumorigenesis and offering potential targets for therapy. Understanding the prevalence, mechanisms, and clinical implications of PIK3CA mutations is essential for developing targeted therapies and improving patient outcomes in EC [42].

The interplay between PTEN and PI3K mutations is pivotal in regulating the PI3K/AKT signaling pathway, which is frequently dysregulated in cancers, including EC. Their association underscores the nuanced control of cellular growth, proliferation, and survival, with implications for oncogenesis [35,44]. When PTEN loss occurs, it lowers the threshold for PI3K pathway activation and primes the pathway for activation but typically requires either growth factors or PI3K mutations. The binding of growth factors to RTKs, such as IGF-1R, HER2, or EGFR, further amplifies PI3K activity and downstream signaling. PI3K mutations hyperactivate the pathway, bypassing the need for external stimuli, thereby driving oncogenesis. PIK3CA mutations frequently co-occur with PTEN loss, amplifying signaling intensity and enhancing oncogenic potential [35]. The dual dysregulation fosters uncontrolled cell proliferation and survival, hallmark features of cancer, by promoting Cyclin D1-mediated cell cycle progression, inhibiting pro-apoptotic factors, and enhancing metabolic reprogramming and angiogenesis [35,44]. Understanding the interplay between PTEN and PI3K mutations highlights critical vulnerabilities in the PI3K/AKT/mTOR pathway. It provides opportunities for therapeutic interventions, such as developing PI3K inhibitors to target PIK3CA-mutant cancers; mTOR inhibitors to act as downstream blockade and counteract PTEN-loss-driven activation; and using combination therapies to target RTKs, PI3K, and AKT synergistically and overcome resistance mechanisms [45].

**Figure 5:**
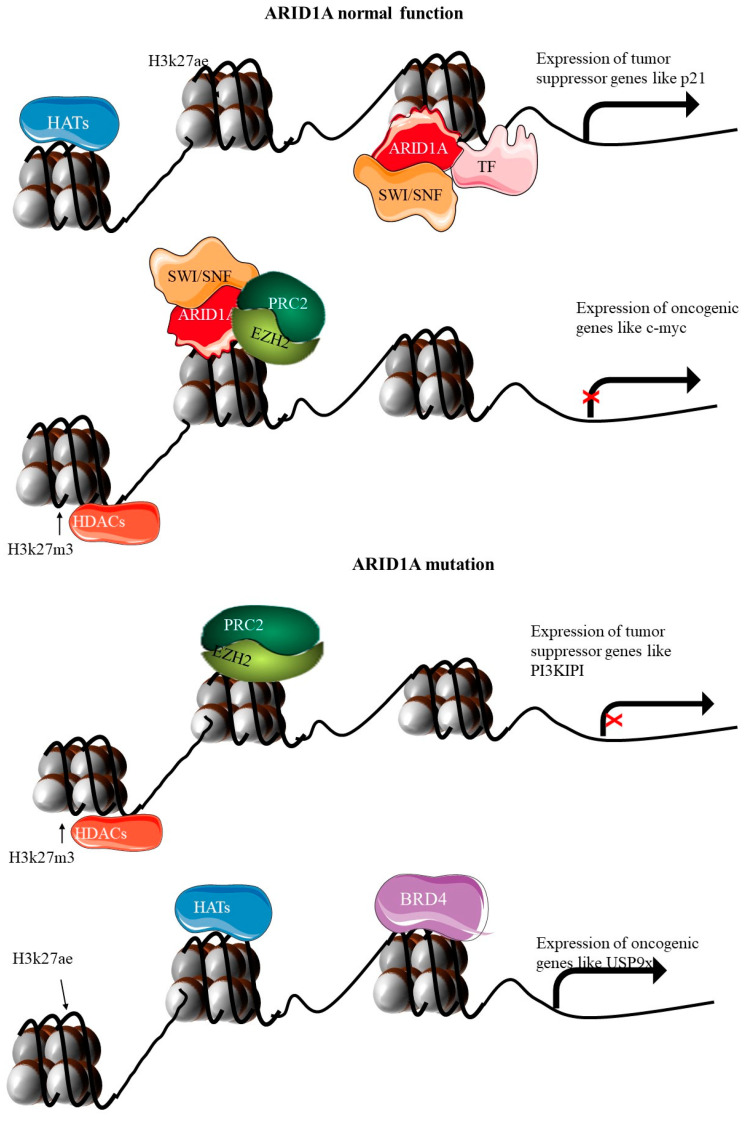
ARID1A mutations in endometrial cancer. ARID1A is a crucial component of the SWI/SNF chromatin remodeling complex, which plays a vital role in regulating gene expression and maintaining genomic stability through chromatin accessibility. Its function intersects with various other epigenetic regulators, including PRC2 (Polycomb Repressive Complex 2), EED (Embryonic Ectoderm Development), HATs (histone acetyltransferases), and HDACs (histone deacetylases). PRC2, composed of core components like EZH2 (catalytic subunit), SUZ12, and EED, is responsible for trimethylating histone H3 at lysine 27 (H3K27me3), a repressive mark that leads to gene silencing. PRC2 plays a critical role in maintaining chromatin in a transcriptionally repressed state. EED, a core PRC2 component, stabilizes PRC2 and assists in recognizing the H3K27me3 mark to propagate repressive signals along chromatin. This ensures heritable gene silencing during cell division. HATs, such as p300/CBP and TIP60, acetylate histone residues, typically at H3K27ac, leading to an open chromatin state conducive to gene transcription. HDACs remove acetyl groups from histone tails, condensing chromatin and repressing gene transcription. They act in opposition to HATs to maintain dynamic chromatin states. In its normal function, ARID1A antagonizes PRC2 activity, promotes histone acetylation through HATs, and limits HDAC-mediated repression. When ARID1A is mutated, this balance is disrupted, leading to excessive PRC2 activity, reduced histone acetylation, and silencing of critical tumor suppressor genes, thereby contributing to oncogenesis.

Among the genetic alterations implicated in EC, mutations in the *ARID1A* gene have garnered significant attention. ARID1A (AT-rich interactive domain-containing protein 1A) is a crucial component of the SWI/SNF chromatin remodeling complex, which plays a vital role in regulating gene expression and maintaining genomic stability [46]. In its normal function, ARID1A antagonizes PRC2 activity, promotes histone acetylation through HATs, and limits HDAC-mediated repression. When ARID1A is mutated, this balance is disrupted, leading to excessive PRC2 activity, reduced histone acetylation, and the silencing of critical tumor suppressor genes, thereby contributing to oncogenesis. Thus, the loss or mutation of ARID1A disrupts these processes, contributing to the pathogenesis of EC. *ARID1A* mutations are found in approximately 30–50% of ECs and are associated with loss of function of the SWI/SNF chromatin remodeling complex, which leads to altered gene expression and contributes to cancer development [46,47]. They are also present in a smaller percentage of non-endometrioid subtypes. These mutations are often characterized by frameshift and nonsense mutations, leading to a truncated, non-functional protein [30,46,48]. Such loss-of-function mutations result in the inactivation of the SWI/SNF complex, thereby affecting chromatin remodeling and transcriptional regulation (shown in Figure 5) [48]. The ARID1A gene plays a pivotal role in various cellular processes, including DNA repair, cell cycle regulation, and apoptosis. The inactivation of ARID1A impairs these critical functions, promoting genomic instability and oncogenic transformation [30]. Specifically, *ARID1A* mutations are associated with defects in the homologous recombination repair pathway, leading to the accumulation of DNA damage and increased mutation rates [48].

In EC, *ARID1A* mutations often co-occur with mutations in other genes such as *PIK3CA* and *PTEN*, suggesting a collaborative role in driving tumorigenesis. The interplay between these mutations exacerbates oncogenic signaling pathways, particularly the PI3K/AKT/mTOR pathway, further promoting cancer cell proliferation and survival [49]. The presence of *ARID1A* mutations in EC has several clinical implications. Studies have shown that *ARID1A* mutations are associated with favorable prognostic outcomes in endometrioid EC, although this association is not universally observed across all studies [48]. The prognostic impact may be influenced by the molecular context and co-occurring genetic alterations. The loss of ARID1A function presents potential therapeutic opportunities. For instance, tumors with ARID1A mutations exhibit increased sensitivity to inhibitors of the ATR and PARP pathways, which are involved in DNA damage response and repair [50]. This synthetic lethality approach exploits the DNA repair deficiencies in ARID1A-mutant cancers, offering a targeted treatment strategy. ARID1A mutations may serve as predictive biomarkers for responses to certain therapies [51,52]. For example, immune checkpoint inhibitors have shown efficacy in ARID1A-mutant tumors, potentially due to the increased mutational burden and neoantigen load associated with these mutations [50,51].

Recent genomic studies have provided deeper insights into the functional consequences of ARID1A mutations and their role in EC. Comprehensive analyses, such as those by The Cancer Genome Atlas (TCGA), have elucidated the molecular subtypes of EC, highlighting the distinct genetic and epigenetic landscapes driven by ARID1A alterations [18,29,52]. Additionally, emerging research is exploring the combination of ARID1A-targeted therapies with other treatment modalities. For instance, combining ATR inhibitors with immune checkpoint blockade is being investigated in preclinical models, showing promising synergistic effects in ARID1A-mutant cancers [52]. Thus, ARID1A mutations are a significant driver of EC, particularly in the endometrioid subtype. These mutations disrupt chromatin remodeling and DNA repair processes, contributing to tumorigenesis. Understanding the prevalence, mechanisms, and clinical implications of ARID1A mutations is essential for developing targeted therapies and improving patient outcomes in EC [29,52].

Chromosomal abnormalities in EC include a wide range of genetic alterations such as copy number variations (CNVs), loss of heterozygosity (LOH), and specific chromosomal rearrangements [14,29]. These abnormalities result in gains and losses of whole chromosomes or large chromosomal regions, as well as structural rearrangements. Common abnormalities include chromosome 1q gains and 16q losses and structural rearrangements [53]. Frequently observed in ECs, these chromosomal alterations are thought to harbor key oncogenes and tumor suppressor genes that drive tumorigenesis [14]. Structural rearrangements include translocations, inversions, and complex rearrangements that can lead to oncogene activation or tumor suppressor gene inactivation. These abnormalities contribute to the pathogenesis and progression of EC by disrupting the critical regulatory pathways involved in cell cycle control, apoptosis, and DNA repair mechanisms [29].

Copy number variations (CNVs), including amplifications and deletions of specific chromosomal regions, are frequently observed in EC [54]. For instance, amplifications of chromosomal regions 1q, 8q, and 10q have been associated with aggressive tumor behavior and poor prognosis [28,55]. Deletions in regions such as 1p, 3p, and 9p are also common and are linked to the inactivation of tumor suppressor genes [54]. Loss of heterozygosity (LOH) is another critical chromosomal alteration observed in EC. LOH at chromosomal regions harboring tumor suppressor genes can lead to the complete inactivation of these genes [55]. Notably, LOH at 10q23, the locus of the PTEN tumor suppressor gene, is frequently reported in endometrial carcinomas and is associated with the development of the endometrioid subtype of EC [55]. Specific chromosomal rearrangements, although less common than CNVs and LOH, also play a role in the pathogenesis of EC [54]. For example, translocations involving chromosomal region 17q21 have been identified in a subset of ECs and are associated with alterations in the expression of the HER2/neu oncogene [56].

The identification of chromosomal abnormalities in EC has significant implications for diagnosis, prognosis, and treatment [54]. Genomic profiling of tumors can help identify patients at higher risk for aggressive disease and guide personalized treatment strategies [52]. Targeted therapies aimed at specific genetic alterations, such as HER2/neu amplification, have shown promise in clinical trials [57]. Hence, chromosomal abnormalities play a crucial role in the development and progression of EC. Understanding these genetic alterations is essential for improving the diagnosis, prognostication, and treatment of EC. Ongoing research and advancements in genomic technologies continue to enhance our knowledge of these abnormalities, offering hope for more effective and personalized therapeutic approaches.

## 4. Classification of Endometrial Cancers into Distinct Molecular Subtypes

**Figure 6:**
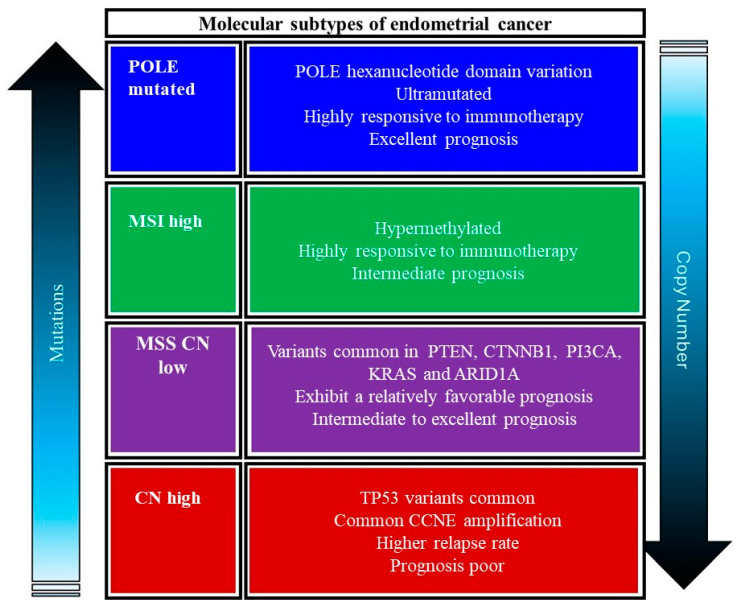
Molecular subtypes of endometrial cancer. The classification of EC into distinct molecular subtypes represents a paradigm shift in the management of this disease. By moving beyond traditional histopathological criteria, this approach offers a more nuanced understanding of the biological diversity of EC, paving the way for more effective, personalized treatment strategies and ultimately improving patient outcomes. POLE mutated is characterized by high mutational burden and distinct prognostic implications due to mutations in the exonuclease domain of the DNA polymerase epsilon (*POLE*) gene. MSI high is a form of genetic hypermutability that results from impaired DNA mismatch repair (MMR) system, leading to high mutation rates. MSS CN low is characterized by few copy number alterations and specific molecular features that distinguish it from other EC subtypes. CN high is marked by a high frequency of copy number alterations, frequent TP53 mutations, and distinct molecular features that contribute to its aggressive clinical behavior. *Abbreviations*: CN—copy number; MSS—microsatellite stable; and MSI—microsatellite instability.

Traditionally, EC has been classified based on histopathological criteria into two main types: Type I (endometrioid) and Type II (non-endometrioid) carcinomas. Type I cancers are typically estrogen-dependent and associated with a better prognosis, while Type II cancers are more aggressive and often present with a poorer prognosis [18]. However, this histological classification does not fully capture the underlying molecular heterogeneity of the disease, leading to variable responses to therapy and outcomes among patients with similar histopathological diagnoses [58]. Advances in genomic technologies have revolutionized our understanding of the molecular landscape of EC. Comprehensive genomic analyses by The Cancer Genome Atlas (TCGA) project have identified four distinct molecular subtypes of EC that provide a more accurate and prognostically relevant classification system: POLE ultramutated, microsatellite instability hypermutated (MSI-H), copy number low, and copy number high (shown in Figure 6) [58]. These molecular subtypes are defined by specific genetic, epigenetic, and transcriptomic alterations, offering insights into the mechanisms driving tumorigenesis and potential therapeutic targets. Each subtype has different mutation profiles and clinical outcomes, guiding personalized treatment strategies [18,29].

### 4.1. POLE-Ultramutated Subtype

POLE-ultramutated EC has garnered significant attention due to its unique genetic profile and clinical behavior. This subtype is characterized by an exceptionally high mutational burden and distinct prognostic implications due to mutations in the exonuclease domain of the DNA polymerase epsilon (*POLE*) gene, which encodes the DNA polymerase epsilon catalytic subunit. These tumors are often associated with a high number of neoantigens, which may enhance immune response [30]. POLE is a key enzyme involved in DNA replication and repair, possessing a proofreading function that corrects replication errors. Mutations in the exonuclease domain of the *POLE* gene (most frequently occurring in exonuclease domain hot spots) disrupt this proofreading ability, leading to an ultramutated phenotype with a high number of single-nucleotide substitutions [59]. These mutations result in a hypermutator phenotype with a significant neoantigen load, which is hypothesized to enhance the immune system’s ability to recognize and attack tumor cells [60].

*POLE*-ultramutated ECs are predominantly endometrioid adenocarcinomas and are often of high grade. Despite their aggressive histopathological appearance, these tumors are associated with a favorable prognosis. The high mutation burden is thought to provoke a robust anti-tumor immune response, contributing to the lower recurrence rates and better overall survival observed in patients with POLE-ultramutated tumors compared to in those with other subtypes [30,61]. The identification of *POLE*-ultramutated EC requires molecular testing. Next-generation sequencing (NGS) is the standard method for detecting POLE mutations and differentiating this subtype from other molecular variants of EC. Immunohistochemistry (IHC) alone is insufficient for diagnosing *POLE* mutations, making NGS an essential tool for accurate classification and personalized treatment planning [30].

Standard treatment for EC typically involves surgery, radiation, and chemotherapy. However, the favorable prognosis of POLE-ultramutated tumors suggests that some patients may benefit from less aggressive treatment strategies. Additionally, the high mutational burden and increased neoantigen presentation in these tumors make them promising candidates for immunotherapy. Early clinical evidence suggests that immune checkpoint inhibitors, such as pembrolizumab, may be particularly effective in treating POLE-ultramutated ECs [16,62]. Ongoing research aims to elucidate the biological mechanisms underlying the favorable outcomes of *POLE*-ultramutated ECs. Studies are also exploring the potential of combining immunotherapy with other treatments to enhance efficacy. Moreover, efforts are being made to develop reliable biomarkers for early detection and monitoring treatment response, which could further improve patient management [32,63]. Thus, *POLE*-ultramutated EC is a unique molecular subtype with distinct genetic and clinical characteristics. The presence of POLE mutations confers a high mutational burden, which is associated with improved prognosis and potential responsiveness to immunotherapy. An accurate molecular diagnosis is crucial for appropriate patient management and optimizing treatment outcomes. As research progresses, there is hope for more targeted and effective therapies that will continue to improve the outlook for patients with this and other subtypes of EC.

### 4.2. Microsatellite Instability (MSI)

Microsatellite instability (MSI) is a form of genetic hypermutability that results from an impaired DNA mismatch repair (MMR) system, leading to high mutation rates [64]. This condition has significant implications in EC, where MSI is an important biomarker for prognosis and therapeutic decision-making. MSI tumors are characterized by a distinct mutational signature and are responsive to immunotherapy [46]. Recent advances in molecular diagnostics and targeted therapies have highlighted the role of MSI in the personalized treatment of EC [65]. Microsatellites are short, repetitive sequences of DNA that are particularly prone to replication errors [64]. The DNA MMR system, which includes proteins such as MLH1, MSH2, MSH6, and PMS2, is responsible for correcting these errors. When this repair system is defective, replication errors accumulate, leading to MSI [64]. In EC, MSI commonly results from epigenetic silencing of the MLH1 gene through promoter hypermethylation, although germline mutations in MMR genes can also contribute, especially in the context of Lynch syndrome [64,65].

MSI is present in approximately 20–30% of all ECs. Tumors with MSI, referred to as being MSI-high (MSI-H), often exhibit distinct pathological and clinical features [64]. MSI-H ECs tend to be of the endometrioid subtype, with a high grade and early-stage presentation [65]. Importantly, MSI-H status is associated with a better overall prognosis compared to microsatellite stable (MSS) tumors, although they may also have a higher likelihood of concurrent Lynch syndrome [66]. The identification of MSI in EC is crucial for guiding treatment and identifying potential Lynch syndrome cases [18,34]. Immunohistochemistry (IHC) is a commonly used screening tool for MMR proteins. Loss of expression of one or more MMR proteins suggests MSI and warrants further genetic testing [65]. Polymerase Chain Reaction (PCR)-based assays are also used to detect MSI by comparing the length of microsatellite sequences in tumor DNA to that in normal DNA [13]. Alternatively, next-generation sequencing (NGS) is used to offer a comprehensive approach to identify MSI and other genetic alterations, providing valuable information for personalized treatment strategies [18,34].

According to [14], MSI status has significant therapeutic implications in EC. In immunotherapy, MSI-H tumors are characterized by a high mutational burden and increased neoantigen load, making them particularly responsive to immune checkpoint inhibitors. Pembrolizumab, an anti-PD-1 antibody, has shown efficacy in treating MSI-H EC, leading to its FDA approval for this indication [2]. Moreover, the role of MSI in predicting chemotherapy response is complex and still under investigation. Some studies suggest that MSI-H tumors may be less responsive to traditional chemotherapeutics, highlighting the need for alternative treatment strategies [14]. The role of MSI in EC is still under research to improve diagnostic and further elucidate therapeutic approaches. The key research focus areas include identifying additional biomarkers that can predict responses to immunotherapy and other treatments; investigating the efficacy of combining immunotherapy with other treatments, such as targeted therapies and radiation; and enhancing genetic counseling and testing for Lynch syndrome in patients with MSI-H tumors to improve early detection and prevention strategies [67]. Thus, MSI is a critical biomarker in EC, influencing prognosis, treatment decisions, and genetic counseling. Advances in molecular diagnostics and targeted therapies continue to improve the management of MSI-H EC, offering hope for better patient outcomes.

### 4.3. Copy-Number-Low Subtype

This subtype, also known as the endometrioid subtype, typically has few copy number alterations and specific molecular features that distinguish it from other EC subtypes. It is often associated with mutations in genes such as *PTEN*, *PIK3CA*, and *CTNNB1*. These tumors have a favorable prognosis and are typically low-grade and early-stage at diagnosis [30]. The CNL subtype of EC exhibits fewer genomic alterations compared to the copy-number-high (CNH) subtype. It is often associated with a lower mutation burden and a more stable genome. This subtype typically includes endometrioid endometrial carcinomas, which are often hormone receptor-positive and exhibit a relatively favorable prognosis [18]. The CNL subtype is frequently characterized by low frequency of somatic copy number alterations; mutations in *PTEN*, *PIK3CA*, and *ARID1A*; and microsatellite stability (MSS). Unlike CNH tumors that exhibit numerous chromosomal amplifications and deletions, CNL tumors maintain a relatively stable genome. The mutations in PTEN, PIK3CA, and ARID1A are common but are not associated with extensive chromosomal instability. CNL tumors typically do not exhibit the high mutation rates associated with microsatellite instability (MSI) [30].

The molecular stability of CNL ECs translates into distinct clinical characteristics that include prognosis, hormone receptor status, and therapeutic strategies. CNL tumors generally have a better prognosis compared to CNH tumors, with lower recurrence rates and higher overall survival [2]. Many CNL tumors are estrogen and progesterone receptor-positive, which may make them more responsive to hormonal therapies [2]. The stable genome of CNL tumors suggests they may respond well to conventional treatments such as surgery and radiation. Hormonal therapies are also a viable option due to the frequent hormone receptor positivity. Targeted therapies directed at the PI3K/AKT/mTOR pathway, given the common mutations in PTEN and PIK3CA, are also being explored [65].

Accurate classification of EC subtypes, including CNL, is crucial for tailoring treatment strategies. Genomic sequencing and immunohistochemistry (IHC) are the two most common diagnostic approaches currently in use. Whole-exome or targeted sequencing is used to identify the specific mutations and copy number alterations characteristic of CNL tumors. IHC for hormone receptors and mismatch repair proteins provide insights into the tumor’s molecular profile and potential subtype [30]. Understanding the CNL subtype has implications for patient care which include biomarker discovery, clinical trials, and long-term outcomes. Identifying the novel biomarkers specific to CNL tumors could enhance diagnostic accuracy and treatment personalization. Investigating the efficacy of targeted therapies and novel agents in CNL EC could expand therapeutic options. Further studies on the long-term outcomes of patients with CNL tumors will help to validate prognostic predictions and inform follow-up strategies [18]. Hence, the CNL subtype of EC represents a distinct molecular and clinical entity with relatively favorable outcomes. Understanding the unique characteristics of CNL tumors can guide more effective and personalized treatment approaches, improving patient prognosis and quality of life.

### 4.4. Copy-Number-High (CNH) Subtype

The copy-number-high (CNH) subtype, also referred to as the serous-like subtype, is marked by a high frequency of copy number alterations, frequent TP53 mutations, and distinct molecular features that differentiate it from other subtypes, contributing to its aggressive clinical behavior [30]. They are often high-grade, aggressive, and associated with poor prognosis. This subtype includes serous and mixed histologies [30]. The CNH subtype of EC is notable for its extensive genomic instability. It is often associated with serous histology but can also be found in high-grade endometrioid carcinomas. The key molecular features of CNH EC include high frequency of somatic copy number alterations (SCNAs); mutations in *TP53*; low frequency of mutations in *PTEN*, *PIK3CA*, and *ARID1A*; and microsatellite stability (MSS). CNH tumors exhibit numerous chromosomal amplifications and deletions, indicating a highly unstable genome [18]. Mutations in the tumor suppressor *gene TP53* are prevalent in CNH tumors, contributing to their aggressive nature and poor prognosis [18]. Unlike the copy-number-low (CNL) subtype, these mutations are less common in CNH tumors. CNH tumors typically do not exhibit microsatellite instability, distinguishing them from the MSI subtype [18]. The molecular instability of CNH ECs translates into distinct clinical characteristics. CNH tumors generally have a poorer prognosis compared to other subtypes, with higher recurrence rates and lower overall survival [2]. Due to the aggressive nature of CNH tumors, treatment often involves a combination of surgery, radiation, and chemotherapy. The efficacy of targeted therapies and immunotherapies is an area of active research [18,29].

Accurate classification of EC is crucial for guiding treatment strategies. Whole-exome or targeted sequencing can identify the specific mutations and extensive SCNAs characteristic of CNH tumors. IHC for p53 protein expression can help identify TP53 mutations and assess tumor subtype [30]. Identifying the novel biomarkers specific to CNH tumors could enhance diagnostic accuracy and treatment personalization. Investigating the efficacy of novel therapies, including targeted agents and immunotherapies, in CNH EC could expand therapeutic options. Further studies on the long-term outcomes of patients with CNH tumors will help validate prognostic predictions and inform follow-up strategies [18]. Thus, the CNH subtype of EC represents a distinct molecular and clinical entity characterized by extensive genomic instability and poor prognosis. Understanding the unique characteristics of CNH tumors can guide more effective and personalized treatment approaches, improving patient outcomes.

## 5. Epigenetic Modifications

**Figure 7:**
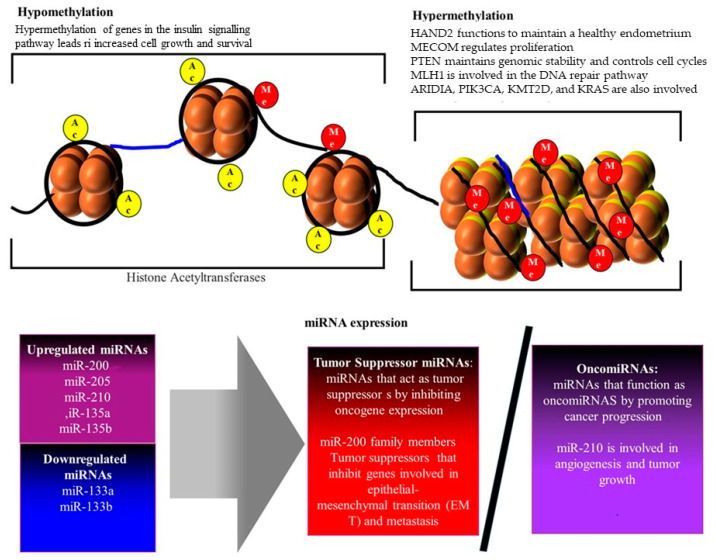
Epigenetic modifications in endometrial cancer. Aberrant DNA methylation patterns, such as hypermethylation of tumor suppressor gene promoters, lead to gene silencing and contribute to carcinogenesis. Hypomethylation of oncogenes can also promote tumor development. Alterations in histone modifications, including methylation, acetylation, and phosphorylation, affect chromatin structure and gene expression. Dysregulation of histone-modifying enzymes has been implicated in EC. MicroRNAs (miRNAs) and long non-coding RNAs (lncRNAs) play significant roles in the post-transcriptional regulation of gene expression. Dysregulation of miRNAs and lncRNAs has been linked to various aspects of EC progression, including cell proliferation, apoptosis, and metastasis.

EC is increasingly recognized as a disease influenced not only by genetic mutations but also by epigenetic modifications. Epigenetic alterations play a critical role in the regulation of gene expression and cancer progression in EC [68]. The modifications, which include DNA methylation, histone modifications, and non-coding RNA expression (shown in Figure 7), play a crucial role in the regulation of gene expression without altering the underlying DNA sequence [28]. Epigenetic changes can drive carcinogenesis by silencing tumor suppressor genes or activating oncogenes, thereby contributing to the development and progression of EC [46].

Recent advances in epigenomic technologies have facilitated the comprehensive mapping of epigenetic changes in EC, revealing distinct epigenetic profiles associated with different molecular subtypes of the disease [69]. These findings have not only enhanced our understanding of the molecular underpinnings of EC but also opened new avenues for targeted therapeutic strategies aimed at reversing aberrant epigenetic modifications [18]. The integration of epigenetic biomarkers into clinical practice holds promise for improving the diagnosis, prognosis, and treatment of EC. Drugs targeting epigenetic modifications are being explored as potential treatments for EC [68].

### 5.1. DNA Methylation in Endometrial Cancer

**Figure 8:**
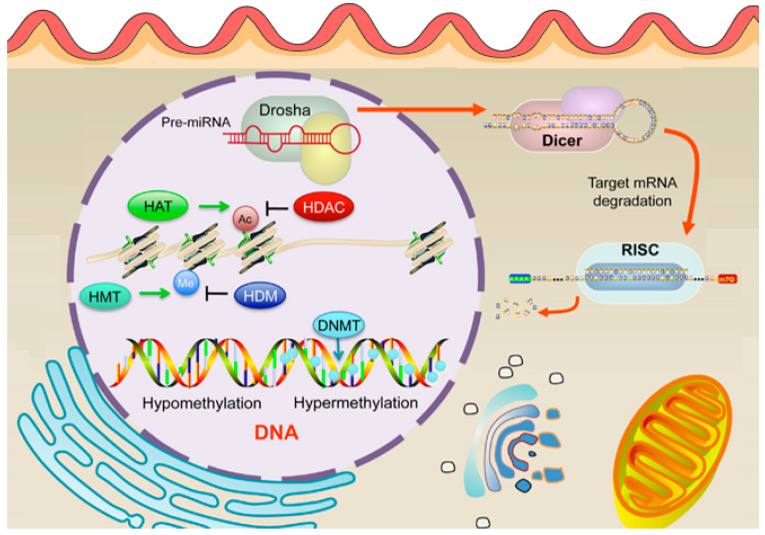
Molecular epigenetic mechanisms of DNA methylation in endometrial cancer. DNA hypermethylation often leads to silencing at CpG islands. The hypermethylated CpG islands normally silence crucial tumor suppressor genes that wreak havoc on the cell’s ability to repair DNA damage, thus hindering its growth and proliferation controls. On the other hand, DNA hypo-methylation is linked to active genes in cancerous cells. It promotes oncogenesis by transcriptional activation of previously silenced oncogenes. It reactivates dormant transposons and induces chromosomal instability at particular pericentromeric satellite regions.

DNA methylation is a crucial epigenetic modification that plays a significant role in gene regulation. In the context of EC, aberrant DNA methylation patterns are common and contribute to the initiation and progression of the disease. This includes hypermethylation of tumor suppressor genes, hypomethylation of oncogenes, and global changes in methylation that affect genomic stability (shown in Figure 8) [46]. DNA methylation typically occurs at the 5-carbon position of cytosine residues within CpG dinucleotides, leading to the formation of 5-methylcytosine. This modification generally acts to repress gene expression by preventing the binding of transcription factors to DNA or by recruiting proteins that mediate gene silencing [46]. In cancer, dysregulation of DNA methylation is a common phenomenon, often resulting in the silencing of key regulatory genes that control cell growth and apoptosis.

In EC, one of the most notable epigenetic changes is the hypermethylation of promoter regions of tumor suppressor genes, leading to their silencing. For example, the *PTEN* gene, which is frequently mutated in EC, is also commonly inactivated through promoter hypermethylation [28]. This loss of PTEN function contributes to the uncontrolled cell proliferation characteristic of cancer. Similarly, the *MLH1* gene, a critical component of the DNA mismatch repair (MMR) system, is often silenced by promoter hypermethylation in microsatellite instability-high (MSI-H) ECs [46]. The loss of MLH1 expression leads to the accumulation of DNA errors, further driving tumorigenesis. While hypermethylation of tumor suppressor genes is a well-recognized phenomenon in EC, global DNA hypomethylation also plays a significant role [70]. Hypomethylation can lead to the activation of oncogenes, such as the insulin-like growth factor 2 (IGF2) gene, which is often overexpressed in EC due to loss of imprinting caused by hypomethylation [46]. Furthermore, hypomethylation of repetitive DNA elements, such as LINE-1, is frequently observed in EC. This can result in chromosomal instability, contributing to the progression of the disease [70].

The alterations in DNA methylation patterns in EC have potential utility as biomarkers for early detection, prognosis, and treatment stratification [18]. For example, the methylation status of the MLH1 promoter is used to identify patients with MSI-H tumors, which have distinct clinical behaviors and may respond differently to immunotherapy [34]. Additionally, global methylation patterns could potentially distinguish between different subtypes of EC, aiding in personalized treatment approaches [46]. The reversible nature of DNA methylation makes it an attractive target for therapeutic intervention. Agents that inhibit DNA methyltransferases (DNMTs), such as 5-azacytidine and decitabine, have shown promise in preclinical models of EC by reactivating silenced tumor suppressor genes and reducing tumor growth [71]. These epigenetic therapies, alone or in combination with other treatments, could offer new avenues for treating EC, particularly in patients with hypermethylated tumors. Thus, DNA methylation plays a critical role in the pathogenesis of EC by modulating the expression of key genes involved in cell cycle regulation, apoptosis, and DNA repair. The aberrant methylation patterns observed in EC not only provide insights into the molecular mechanisms driving the disease but also offer potential biomarkers for diagnosis and targets for therapy. Continued research in this area is likely to yield further advances in our understanding and treatment of EC.

### 5.2. Histone Modifications in Endometrial Cancer

**Figure 9:**
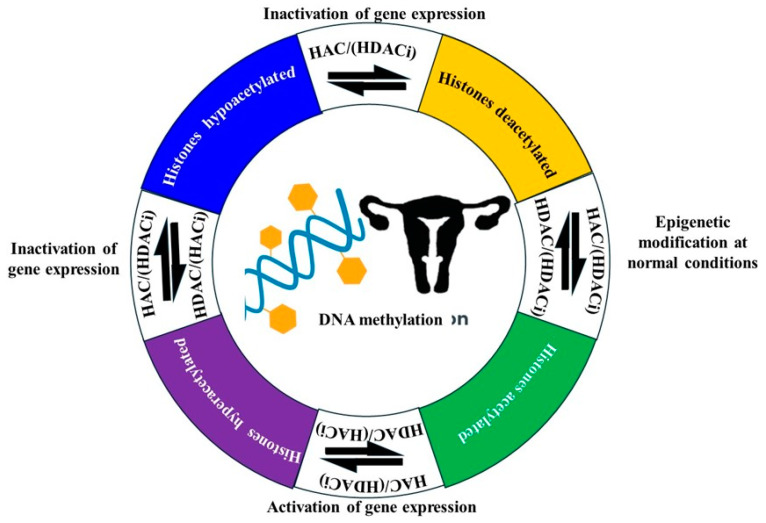
Histone modification in endometrial cancers. Hypoacetylations on tumor suppressor gene sequences or hyperacetylations on oncogene areas are among the common aberrant histone modifications in endometrial carcinogenesis. Enzymatic inhibitors can biochemically rectify these processes. Tumor suppressor gene deacetylation and reactivation can be prevented by employing histone deacetylase inhibitors (HDACis). Similarly to this, acetyltransferase inhibitors (HACis) are potential drugs for EC treatments since they can prevent oncogenes from being acetylated and inactivated.

Histone modifications are critical epigenetic regulators that influence chromatin structure and gene expression, playing a significant role in the development and progression of EC [68]. These modifications, which include acetylation, methylation, phosphorylation, ubiquitination, and sumoylation of histone tails (shown in Figure 9), can either activate or repress transcriptional activity depending on the specific residues modified and the type of modification [18]. In the context of EC, aberrant histone modifications have been increasingly recognized as key factors contributing to tumorigenesis. One of the most studied histone modifications in EC is histone methylation. Methylation of histone H3 on lysine residues 4 (H3K4), 9 (H3K9), and 27 (H3K27) is particularly relevant, as these marks are associated with either active or repressive chromatin states [18]. For instance, H3K4 methylation is typically linked with transcriptional activation, while *H3K27* methylation is associated with gene repression. Studies have shown that alterations in the enzymes responsible for adding or removing these methyl marks, such as the *H3K27* methyltransferase EZH2, are often dysregulated in EC [68]. Overexpression of EZH2, leading to increased H3K27 trimethylation (*H3K27me3*), has been correlated with poor prognosis and aggressive tumor behavior [68,72].

Histone acetylation, another important modification, is generally associated with transcriptional activation due to the relaxation of chromatin structure, allowing transcription factors access to DNA [68]. Dysregulation of histone acetyltransferases (HATs) and histone deacetylases (HDACs) have been implicated in EC, with alterations in these enzymes leading to an imbalance in acetylation patterns that can either promote or inhibit tumor growth [18,68]. HDAC inhibitors, which restore normal acetylation levels, have shown promise in preclinical models of EC by reactivating tumor suppressor genes and inducing cancer cell death [73]. Moreover, recent research has identified the interplay between histone modifications and other epigenetic mechanisms, such as DNA methylation and non-coding RNAs, in the regulation of gene expression in EC [18]. This crosstalk contributes to the complex epigenetic landscape of the disease and underscores the potential for therapeutic interventions targeting multiple epigenetic pathways [68]. Thus, histone modifications play a crucial role in the epigenetic regulation of EC. Understanding these modifications provides insights into the molecular mechanisms driving the disease and highlights potential targets for novel therapeutic strategies aimed at reversing aberrant epigenetic changes.

## 6. Genomic Techniques and Approaches

The exploration of the genomic landscape of EC has been significantly advanced by the development and application of various genomic techniques. These techniques have enabled a deeper understanding of the molecular underpinnings of EC, leading to more precise diagnostic tools, the identification of novel therapeutic targets, and the development of personalized treatment strategies [14]. The implementation of genomic techniques such high-throughput sequencing (HTS) technologies, bioinformatics, and computational tools has not only facilitated the molecular subtyping of EC but also revealed the complex interplay of the genetic, epigenetic, and environmental factors contributing to tumor development and progression [18,74].

### 6.1. High-Throughput Sequencing Technologies

High-throughput sequencing (HTS) technologies have revolutionized cancer genomics, offering unprecedented insights into the molecular landscape of various malignancies, including EC [18,29]. HTS technologies, including whole-genome sequencing (WGS), whole-exome sequencing (WES), and RNA sequencing (RNA-seq), have been pivotal in uncovering these alterations, leading to the identification of distinct molecular subtypes of EC with differing prognoses and therapeutic responses. The application of HTS has also facilitated the discovery of novel biomarkers and therapeutic targets, paving the way for more personalized and effective treatment strategies [74].

### 6.2. Next-Generation Sequencing (NGS)

Next-generation sequencing (NGS) has emerged as a transformative tool in the study of EC, significantly advancing our understanding of its molecular underpinnings [75]. NGS technologies have been pivotal in identifying mutations, copy number variations, and other genetic alterations in EC. It offers high sensitivity, speed, and the ability to detect a wide range of genetic alterations, including single-nucleotide variants (SNVs), insertions, deletions, and structural variants [18]. NGS allows for comprehensive profiling of these alterations, providing insights that are crucial for diagnosis, prognosis, and the development of targeted therapies. It has been instrumental in identifying key mutations, gene expression changes, and structural variations in EC. For example, studies utilizing whole-exome sequencing (WES) have uncovered frequent mutations in genes such as *PTEN*, *PIK3CA*, *ARID1A*, and *CTNNB1*, which are involved in the critical signaling pathways regulating cell growth and apoptosis [75]. NGS has facilitated the classification of EC into distinct molecular subtypes, such as the POLE-ultramutated, microsatellite instability (MSI), copy-number-low, and copy-number-high subtypes. These molecular subtypes have been shown to have significant prognostic and therapeutic implications [65].

Furthermore, NGS has enabled the identification of potential biomarkers for targeted therapies. For instance, the detection of ERBB2 amplifications and PIK3CA mutations through NGS has led to the exploration of targeted inhibitors in clinical trials [18]. NGS is also pivotal in understanding the mechanisms of resistance to therapies, allowing for the adaptation of treatment strategies based on the evolving molecular landscape of the tumor [75]. The use of NGS in EC research continues to evolve, with ongoing studies aiming to integrate genomic data with other omics approaches, such as proteomics and metabolomics, to provide a more comprehensive understanding of tumor biology. As NGS technologies become more accessible and cost-effective, their integration into clinical practice is expected to further personalize the management of EC, improving outcomes for patients [65].

### 6.3. Whole-Exome and Whole-Genome Sequencing

Whole-exome sequencing (WES) and whole-genome sequencing (WGS) have significantly contributed to the understanding of the genetic landscape of EC. These high-throughput sequencing technologies enable comprehensive analysis of genetic alterations, offering insights into the mutational profiles and potential therapeutic targets in EC [46,75]. WES focuses on sequencing the protein-coding regions of the genome, which constitute about 1–2% of the human genome but harbor approximately 85% of disease-related variants [32]. In EC, WES has been instrumental in identifying recurrent mutations in key driver genes. For instance, studies have consistently found mutations in genes such as *PTEN*, *PIK3CA*, *ARID1A*, *CTNNB1*, and *TP53* in various subtypes of EC. These genes are involved in critical pathways like the PI3K/AKT/mTOR pathway, WNT/β-catenin signaling, and chromatin remodeling, which are crucial for cell proliferation and survival [65]. WES has also been pivotal in the molecular classification of EC, as demonstrated by The Cancer Genome Atlas (TCGA) project. This large-scale study classified ECs into four distinct molecular subtypes: POLE ultramutated, microsatellite instability (MSI) hypermutated, copy number low, and copy number high (serous-like). Each subtype is associated with unique genetic alterations and clinical outcomes, highlighting the heterogeneity of the disease and guiding personalized treatment approaches [29].

WGS, which sequences the entire genome, provides a more comprehensive analysis by capturing not only coding but also non-coding regions, structural variants, and other regulatory elements [32]. This approach has revealed novel insights into the genomic architecture of EC, including the identification of non-coding mutations and structural rearrangements that may play a role in tumorigenesis [65]. One significant finding from WGS studies is the identification of structural variations such as copy number alterations and gene fusions that are not typically detected by WES. For example, ERBB2 amplifications, which are more common in serous endometrial carcinomas, have been detected through WGS and are associated with poor prognosis and potential responsiveness to HER2-targeted therapies [29]. Additionally, WGS has uncovered complex rearrangements and non-coding region mutations that may contribute to gene dysregulation in EC. Furthermore, WGS has been useful in identifying mutational signatures associated with specific DNA repair defects, such as those resulting from defects in the mismatch repair (MMR) system, which are prevalent in MSI-high ECs. Understanding these signatures can inform the use of targeted therapies, such as immune checkpoint inhibitors, which have shown efficacy in MSI-high tumors [32].

The integration of WES and WGS into clinical practice has the potential to revolutionize the management of EC. These technologies provide a more detailed molecular profile of tumors, enabling the identification of actionable mutations and the development of targeted therapies. For instance, the identification of *PIK3CA* mutations has led to the exploration of PI3K inhibitors in clinical trials. Moreover, the ability of WGS to detect structural variants and non-coding mutations may uncover new therapeutic targets and biomarkers for patient stratification. As sequencing technologies continue to advance, the cost of WES and WGS is expected to decrease, making these approaches more accessible in clinical settings. Future research will likely focus on integrating genomic data with other omics approaches, such as transcriptomics and proteomics, to gain a more holistic understanding of EC and to refine therapeutic strategies.

### 6.4. RNA Sequencing (RNA-Seq)

RNA sequencing (RNA-seq) has emerged as a powerful tool for transcriptome analysis, enabling a comprehensive understanding of gene expression profiles, alternative splicing events, and non-coding RNA activity in various cancers, including EC [76]. This technology provides insights into the molecular mechanisms driving EC pathogenesis, progression, and treatment resistance, offering opportunities for the development of novel therapeutic strategies and biomarkers. RNA-seq has been instrumental in delineating the molecular subtypes of EC, which have distinct gene expression profiles and clinical outcomes [76]. The Cancer Genome Atlas (TCGA) initially identified four molecular subtypes of EC: POLE ultramutated, microsatellite instability (MSI) hypermutated, copy number low (endometrioid), and copy number high (serous-like) using a combination of RNA-seq and other genomic techniques [77]. These subtypes have since been validated and refined through RNA-seq studies, which have provided deeper insights into the transcriptional landscapes associated with each subtype. For example, the POLE-ultramutated subtype is characterized by high expression of immune-related genes, reflecting an immune-rich tumor microenvironment that may be amenable to immunotherapy [76].

Conversely, the serous-like subtype shows high expression of genes involved in cell cycle regulation and DNA damage response, highlighting potential targets for therapeutic intervention, such as PARP inhibitors [63]. RNA-seq has also uncovered the complexity of alternative splicing events in EC, revealing novel isoforms that may contribute to tumorigenesis and drug resistance. For instance, a recent study by Li and co-workers identified a set of differentially spliced genes in EC that are associated with key oncogenic pathways, including the PI3K/AKT/mTOR and Wnt/β-catenin pathways [78]. These splicing events could serve as potential biomarkers for disease progression and treatment response. In addition to mRNA transcripts, RNA-seq has shed light on the role of non-coding RNAs (ncRNAs), such as microRNAs (miRNAs) and long non-coding RNAs (lncRNAs), in EC. For example, Zhang and co-workers reported the upregulation of the lncRNA HOTAIR in EC tissues, which was associated with poor prognosis and enhanced metastatic potential [79]. Similarly, miRNA profiling through RNA-seq has identified miRNAs, such as miR-200c and miR-205, that are differentially expressed in EC and may regulate epithelial-to-mesenchymal transition (EMT) and other critical processes [80].

RNA-seq has provided significant insights into the tumor microenvironment (TME) of EC, particularly in understanding the immune landscape. Recent RNA-seq studies have identified distinct immune-related gene expression signatures that correlate with patient outcomes and response to immunotherapy [81]. For instance, tumors with high expression of PD-L1 and other immune checkpoint molecules have been shown to respond better to checkpoint inhibitors, underscoring the potential of RNA-seq in guiding personalized immunotherapy strategies [82]. Moreover, RNA-seq has been used to profile the TME, revealing the presence of various immune cell subsets, such as tumor-infiltrating lymphocytes (TILs) and macrophages, which play critical roles in tumor progression and immune evasion [83]. Understanding the transcriptional profiles of these immune cells through RNA-seq can inform the development of combination therapies that target both the tumor cells and the immune components of the TME [32].

The insights gained from RNA-seq studies in EC have significant clinical implications. By identifying distinct molecular subtypes, alternative splicing events, and non-coding RNAs, RNA-seq has the potential to improve patient stratification, guide therapeutic decisions, and identify novel targets for drug development. For example, the identification of immune-related gene expression signatures through RNA-seq can inform the use of immunotherapies in patients with specific molecular subtypes of EC [76]. Looking forward, the integration of RNA-seq with other omics technologies, such as proteomics and single-cell RNA-seq, will provide a more comprehensive understanding of the molecular and cellular heterogeneity in EC. This multi-omics approach is expected to uncover novel therapeutic targets and biomarkers, ultimately improving patient outcomes.

### 6.5. Bioinformatics and Computational Tools

The integration of bioinformatics and computational tools in EC research has significantly advanced our understanding of the disease’s molecular underpinnings [84]. These tools have enabled the discovery of novel biomarkers, molecular subtypes, and therapeutic targets, paving the way for personalized medicine [85]. As bioinformatics continues to evolve, it holds great potential for further breakthroughs in EC research, ultimately improving patient outcomes and guiding clinical decision-making [84].

### 6.6. Algorithms for Mutation Detection and Interpretation

The analysis of NGS data requires sophisticated bioinformatics tools to accurately detect and interpret genetic mutations [86]. Detecting and interpreting these mutations are critical steps in understanding the molecular basis of EC and in the development of personalized treatment strategies [15]. Advances in sequencing technologies have led to the generation of vast amounts of genomic data, necessitating the use of sophisticated algorithms for the accurate detection and interpretation of mutations [87]. These algorithms help in identifying driver mutations, predicting their functional impacts, and linking them to therapeutic options, thereby enhancing the precision of cancer management [86]. The raw data generated through genomic profiling using the high-throughput sequencing technologies require rigorous processing and analysis to identify mutations accurately. Algorithms such as MuTect, VarScan, and the GATK are widely used for calling somatic mutations from sequencing data [67]. Algorithms for mutation detection typically involve several steps: read alignment, variant calling, and post-processing to filter out false positives [86]. Tools like ANNOVAR and SnpEff help annotate and predict the functional impact of identified mutations, aiding in the understanding of their potential role in cancer [87].

The GATK (Genome Analysis Toolkit) is one of the most widely used tools for variant calling in cancer genomics. It incorporates a series of best practices for read alignment, base quality score recalibration, and variant calling, which are essential for identifying somatic mutations in tumor samples [88]. The GATK has been successfully applied in various studies on EC, enabling the identification of both common and rare mutations associated with the disease [86]. Another powerful tool is MuTect2, which is specifically designed to detect somatic point mutations in paired tumor–normal samples. This tool is particularly effective in identifying low-frequency mutations that might be overlooked by other methods [87]. In the context of EC, MuTect2 has been instrumental in uncovering mutations in key genes such as PTEN, PIK3CA, and ARID1A, which are frequently altered in this cancer type [89]. Strelka2 is another mutation caller that has gained prominence for its sensitivity and accuracy in detecting both single-nucleotide variants (SNVs) and small insertions/deletions (indels) [90]. Strelka2’s efficiency in handling high-depth sequencing data makes it a valuable tool for analyzing complex tumor genomes, including those of EC [87].

Once mutations are detected, the next challenge is to interpret their potential impact on protein function and their role in cancer. This interpretation is crucial for distinguishing between driver mutations, which contribute to cancer progression, and passenger mutations, which are biologically neutral [87]. OncoKB is a precision oncology knowledge base that provides curated information about the oncogenic effects and treatment implications of specific mutations [67]. By integrating genomic data with OncoKB, researchers and clinicians can classify mutations in EC into categories such as likely oncogenic, tumor suppressor, or unknown significance, thereby aiding in clinical decision-making [67,91]. CHASM (Cancer-Specific High-Throughput Annotation of Somatic Mutations) is an algorithm designed to predict the likelihood that a given mutation is a driver mutation. It uses machine learning models trained on large datasets of known cancer mutations and can accurately prioritize mutations in EC for further functional validation [15,87].

Polymorphism Phenotyping v2 (PolyPhen-2) is another widely used tool that predicts the potential impact of an amino acid substitution on the structure and function of a protein. While not cancer-specific, PolyPhen-2 has been applied to the interpretation of mutations in EC, particularly in the context of identifying deleterious variants in tumor suppressor genes [92]. Rare Exome Variant Ensemble Learner (REVEL) is a meta-predictor that combines multiple algorithms to predict the pathogenicity of rare variants [93]. REVEL’s application in EC has been useful for assessing the clinical significance of novel or rare mutations, providing insights into their potential role in cancer development [93,94]. The integration of these mutation detection and interpretation algorithms into clinical workflows is critical for the advancement of personalized medicine in EC [93]. Emerging tools that combine the strengths of multiple algorithms and incorporate additional data types, such as epigenetic marks and transcriptomic profiles, are likely to improve the accuracy and clinical utility of mutation analysis. Additionally, the development of cloud-based platforms that facilitate the sharing and analysis of genomic data across institutions is expected to enhance collaborative efforts in EC research. Such platforms will enable the continuous refinement of existing algorithms and the creation of new tools tailored to the unique genomic landscapes of different cancer types, including EC.

### 6.7. Databases and Resources for Genomic Data

The integration and interpretation of genomic data are facilitated by various databases and resources. The Cancer Genome Atlas (TCGA) provides a comprehensive dataset of genomic, epigenomic, transcriptomic, and proteomic data across various cancer types, including EC [46]. The Catalogue of Somatic Mutations in Cancer (COSMIC) is a database that catalogs somatic mutations in human cancers, providing valuable information for the study of EC [93,95]. The vast amount of data generated by NGS requires significant computational resources and expertise to process, analyze, and interpret [32]. Distinguishing between driver mutations and passenger mutations remains a challenge, requiring robust functional assays and validation studies [93]. The quality and purity of tumor samples can affect the accuracy of sequencing results, with tumor heterogeneity and contamination by normal tissue posing additional challenges [96]. Despite advances in sequencing technologies, certain regions of the genome remain difficult to sequence and interpret accurately [93]. Understanding the biological significance of many genetic alterations, especially those in non-coding regions, remains limited, necessitating further functional studies [87]. The translation of genomic findings into clinical practice is still in its infancy, with challenges in integrating genomic data into standard care protocols and ensuring accessibility to precision medicine [32].

## 7. Clinical Implications of Genomic Findings

The genomic landscape of EC has provided valuable insights that have significant clinical implications [1]. The translation of these genomic discoveries into clinical practice has the potential to revolutionize the management of EC through personalized medicine, the development of targeted therapies, and the identification of prognostic and predictive biomarkers [15]. Table 1 summarizes the application of the genomic underpinnings of EC in current and future clinical practice, highlighting the type of knowledge, study orders, techniques, and clinical implications. The incorporation of genomic underpinnings into EC clinical practice demonstrates the transformative potential of precision medicine. Molecular profiling, particularly through NGS and MSI testing, is now integral for guiding therapeutic decisions. Future advancements will likely expand the repertoire of actionable biomarkers, offering hope for improved outcomes in EC management [12,67]. Advances in genomic research have identified various genetic alterations in EC that can be targeted by specific therapies. For instance, mutations in the PI3K/AKT/mTOR pathway, which are prevalent in EC, have led to the development of targeted inhibitors for this pathway. Targeted therapies aimed at the PI3K/AKT/mTOR pathway have shown promise in treating EC [2]. The presence of mutations in genes such as *PTEN, PIK3CA*, and *ARID1A* makes this pathway a critical target for intervention [19]. Several targeted therapies have been developed based on the genomic alterations identified in EC, leading to more personalized treatment approaches. Alpelisib, a PI3K inhibitor, has shown efficacy in treating patients with *PIK3CA*-mutated cancers, including EC [97]. Everolimus, an mTOR inhibitor, has demonstrated activity in EC, particularly in tumors with *PTEN* mutations [19].

The identification of genomic biomarkers is crucial for predicting disease prognosis and treatment responses in EC. Microsatellite instability high (MSI-H) and deficient mismatch repair (dMMR) are biomarkers associated with better responses to immunotherapy in EC [1]. Pembrolizumab, an immune checkpoint inhibitor, has been approved for MSI-H/dMMR ECs [16]. TP53 mutations are indicative of high-grade, aggressive EC and are associated with poor prognosis, helping stratify patients for more aggressive treatment regimens [58]. Several clinical trials are ongoing to evaluate the efficacy of targeted therapies and the use of biomarkers in EC. A phase II trial (NCT03367741) is investigating the combination of PI3K inhibitors with standard chemotherapy in PIK3CA-mutated EC [2]. The NCT02725268 trial is evaluating the efficacy of pembrolizumab in MSI-H/dMMR EC patients, showing promising results in terms of response rates and progression-free survival [58].

One of the significant challenges in translating genomic data into clinical practice is the variability in patient responses to targeted therapies [1]. EC exhibits significant intra-tumoral and inter-tumoral heterogeneity, leading to variable responses to the same targeted therapies [19]. The development of resistance to targeted therapies poses a challenge, necessitating combination therapies or the development of next-generation inhibitors [98]. The integration of genomic data into routine clinical workflows involves several logistical and practical challenges. Implementing comprehensive genomic profiling in clinical practice requires significant resources, including access to advanced sequencing technologies and bioinformatics support [58]. Effective integration of genomic data necessitates collaboration among oncologists, geneticists, pathologists, and bioinformaticians to interpret the data and make informed treatment decisions [97].

## 8. Future Directions in Genomic Research and Clinical Applications

CRISPR-Cas9 technology has revolutionized genomic research by enabling precise, targeted modifications to the DNA of living organisms [49]. In the context of EC, CRISPR offers the potential to create specific gene knockouts or modifications to study the function of genes involved in cancer development and progression [79]. For instance, CRISPR can be used to disrupt oncogenes or repair tumor suppressor genes in cellular models, providing insights into their roles and offering pathways for therapeutic intervention [49]. Moreover, advancements in CRISPR technology, such as CRISPR-Cas12 and CRISPR-Cas13, have expanded the toolkit for genomic editing and RNA targeting, respectively, broadening the scope of cancer research [49,79]. Single-cell genomics has emerged as a powerful tool to dissect the heterogeneity of tumor cells at an unprecedented resolution [79]. By analyzing the genetic and transcriptomic profiles of individual cells within a tumor, researchers can uncover subpopulations of cancer cells that contribute to treatment resistance and disease recurrence [99]. Spatial transcriptomics further complement this by mapping gene expression within the tissue context, preserving spatial information that is lost in traditional bulk sequencing approaches [83]. These technologies enable a more comprehensive understanding of the tumor microenvironment and the interactions between cancer cells and their surrounding stromal and immune cells, which is crucial for developing targeted therapies.

The integration of multi-omics data is a burgeoning field that seeks to combine different layers of biological information to provide a holistic view of disease mechanisms. In EC, combining genomic data (e.g., DNA mutations, copy number variations) with transcriptomic (mRNA expression), proteomic (protein expression and modifications), and metabolomic (metabolic pathways) data can reveal complex regulatory networks and identify key drivers of cancer progression [100]. For example, proteogenomics integrates proteomic and genomic data to connect genomic alterations to protein expression changes, which can be more directly linked to phenotypic outcomes [101]. A multi-omics approach enables the identification of biomarkers that might not be apparent when examining a single omics layer. For instance, post-translational modifications of proteins, which are critical in cancer signaling pathways, can be missed by genomic or transcriptomic analyses alone [85]. Integrating these data types enhances the understanding of the molecular underpinnings of EC and aids in the identification of novel therapeutic targets. Additionally, it can improve patient stratification for personalized treatments by considering a more comprehensive molecular profile [101].

The ultimate goal of genomic research in EC is to facilitate the development of personalized treatment plans. Precision oncology aims to tailor treatments based on the specific genetic and molecular profile of a patient’s tumor. This approach can lead to the selection of therapies that are more effective and have fewer side effects compared to traditional one-size-fits-all treatments [83]. Advances in high-throughput sequencing and bioinformatics have enabled the identification of actionable mutations and the development of targeted therapies, such as PI3K/AKT/mTOR inhibitors for patients with relevant pathway alterations [18]. Despite the promise of personalized medicine, there are significant ethical and practical challenges to its widespread implementation. One major concern is equitable access to genomic testing and targeted therapies, which can be expensive and limited to specialized centers [83]. Additionally, the interpretation of genomic data requires sophisticated bioinformatics tools and expertise, which may not be readily available in all healthcare settings. Ethical issues also arise regarding genetic privacy and the potential for genetic discrimination. Ensuring that patients understand the implications of genomic testing and maintaining confidentiality is paramount [32].

## 9. Conclusions

The intricate genomic landscape of EC and its profound implications for clinical practice form the foundation of our understanding of this malignancy. Key genetic alterations, such as mutations in *PTEN*, *PIK3CA*, and *ARID1A,* as well as chromosomal abnormalities are a tremendously important therapeutic arsenal that may extrapolate to highly protracted remissions and curative therapies for EC. The identification of distinct molecular subtypes, including POLE ultramutated, MSI, copy number low, and copy number high, underscores the heterogeneity of EC and highlights the necessity of tailored therapeutic approaches. Emerging technologies, such as CRISPR gene editing, single-cell genomics, and spatial transcriptomics, have significantly advanced our ability to study cancer at an unprecedented resolution. These innovations, coupled with the integration of multi-omics data—encompassing genomics, transcriptomics, proteomics, and metabolomics—offer a holistic view of the disease, enhancing our ability to identify novel biomarkers and therapeutic targets.

The translation of genomic findings into personalized medicine and precision oncology is increasingly becoming a reality in clinical practice. The development of targeted therapies based on specific genetic alterations, such as PI3K/AKT/mTOR inhibitors, exemplifies the potential for improved treatment efficacy and reduced side effects. However, the journey from bench to bedside is fraught with challenges, including the variability in patient responses, the integration of genomic data into clinical workflows, and the ethical considerations surrounding genetic testing and personalized treatments. Looking ahead, the ongoing need for research and collaboration remains paramount. The complexities of EC demand a multidisciplinary approach, leveraging expertise from genetics, bioinformatics, clinical medicine, and beyond. By fostering collaboration and continuing to push the boundaries of genomic research, we can look forward to future breakthroughs in the diagnosis and treatment of EC. There is optimism that with sustained effort, we can achieve more precise, effective, and personalized care for patients, ultimately improving outcomes and quality of life for those affected by this disease.

## Figures and Tables

**Table 1:** Genomic applications in clinical practice.

Application	Study to Order	Technique Used	Clinical Implication	Status	Reference
Mismatch Repair Status	Immunohistochemistry (IHC), PCR	IHC for MLH1, MSH2, MSH6, PMS2; MSI PCR	Therapeutic: response to checkpoint inhibitors	Current	[30,32]
TP53 Mutation	Next-Generation Sequencing (NGS)	NGS or Sanger Sequencing	Prognostic: poor outcomes in serous EC	Current	[58]
POLE Mutation	NGS	Whole-Exome or Targeted Sequencing	Prognostic: favorable survival	Current	[30]
PIK3CA/PTEN Mutations	NGS	Targeted Gene Panels	Therapeutic: PI3K inhibitors	Current	[32]
ARID1A Mutations	NGS	Targeted Sequencing	Therapeutic: potential use of PARP inhibitors	Emerging	[48]
HER2 Amplifications	FISH, IHC	Fluorescence In Situ Hybridization (FISH)	Therapeutic: HER2-targeted therapies	Future	[57]
KRAS Mutations	NGS	Targeted Gene Panels	Therapeutic: potential KRAS inhibitors	Future	[65]
Epigenetic Profiling	Bisulfite Sequencing	Methylation Arrays	Diagnostic/prognostic: early detection	Future	[68]

## Data Availability

Data sharing is not applicable.
